# Characterization of Mobile Genetic Elements Using Long-Read Sequencing for Tracking *Listeria monocytogenes* from Food Processing Environments

**DOI:** 10.3390/pathogens9100822

**Published:** 2020-10-07

**Authors:** Hee Jin Kwon, Zhao Chen, Peter Evans, Jianghong Meng, Yi Chen

**Affiliations:** 1 Department of Nutrition and Food Science, Joint Institute for Food Safety and Applied Nutrition, Center for Food Safety and Security Systems, University of Maryland, College Park, MD 20740, USA; hkwon22@umd.edu (H.J.K.); zhchen29@umd.edu (Z.C.); jmeng@umd.edu (J.M.); 2 Division of Microbiology, Office of Regulatory Science, Center for Food Safety and Applied Nutrition, U.S. Food and Drug Administration, College Park, MD 20740, USA; peter.evans@usda.gov

**Keywords:** *Listeria monocytogenes*, nanopore long-read sequencing, mobile genetic elements, evolutionary analysis, whole-genome sequencing

## Abstract

Recently developed nanopore sequencing technologies offer a unique opportunity to rapidly close the genome and to identify complete sequences of mobile genetic elements (MGEs). In this study, 17 isolates of *Listeria monocytogenes* (*Lm*) epidemic clone II (ECII) from seven ready-to-eat meat or poultry processing facilities, not known to be associated with outbreaks, were shotgun sequenced, and among them, five isolates were further subjected to long-read sequencing. Additionally, 26 genomes of *Lm* ECII isolates associated with three listeriosis outbreaks in the U.S. and South Africa were obtained from the National Center for Biotechnology Information (NCBI) database and analyzed to evaluate if MGEs may be used as a high-resolution genetic marker for identifying and sourcing the origin of *Lm*. The analyses identified four *comK* prophages in 11 non-outbreak isolates from four facilities and three *comK* prophages in 20 isolates associated with two outbreaks that occurred in the U.S. In addition, three different plasmids were identified among 10 non-outbreak isolates and 14 outbreak isolates. Each *comK* prophage and plasmid was conserved among the isolates sharing it. Different prophages from different facilities or outbreaks had significant genetic variations, possibly due to horizontal gene transfer. Phylogenetic analysis showed that isolates from the same facility or the same outbreak always closely clustered. The time of most recent common ancestor of the *Lm* ECII isolates was estimated to be in March 1816 with the average nucleotide substitution rate of 3.1 × 10^−7^ substitutions per site per year. This study showed that complete MGE sequences provide a good signal to determine the genetic relatedness of *Lm* isolates, to identify persistence or repeated contamination that occurred within food processing environment, and to study the evolutionary history among closely related isolates.

## 1. Introduction

Listeriosis, a foodborne illness caused by *Listeria monocytogenes* (*Lm*), is a rare disease but can be severe. Patients can develop septicemia, meningitis, encephalitis, among other conditions and in severe cases, die. Among the most common foodborne illnesses, listeriosis exhibits the highest mortality rate approaching 30%, with pregnant women, newborn infants, and the immunocompromised population at the highest risk [1]. Due to its robust viability under a wide range of pH, temperature, and salinity, *Lm* is remarkably ubiquitous in the food processing environments and may persist up to several years [2]. Even though invasive listeriosis infections are not common in healthy individuals, it is important to understand the potential public health impact of *Lm* contamination [1]. Strains from many listeriosis outbreaks belong to a small number of clones, designated as epidemic clones (ECs) or clonal complexes (CCs) [3]. Among the ECs, epidemic clone II (ECII), also known as clonal complex 6 (CC6), was first recognized in a 1998 multistate listeriosis outbreak associated with contaminated hot dogs in the United States [4]. In 2002, another multistate listeriosis outbreak caused by ECII strains occurred, which was associated with contaminated turkey deli products [5]. More recently, *Lm* ECII was implicated in a major listeriosis outbreak in South Africa from 2017 to 2018 due to the contamination of ready-to-eat (RTE) meat products [6]. It was the world’s largest listeriosis outbreak with a total of 1060 cases [6]. Understanding the contamination, persistence, and transmission of *Lm* ECII in food processing facilities is important to develop preventive control measures against this pathogen. Following the 2002 listeriosis outbreak in the U.S., additional *Lm* ECII strains from RTE meat or poultry processing facilities were recovered during regular surveillance [7]. Isolates from each facility were collected from two months apart to 22 months apart, and some isolates were analyzed by polymerase chain reaction (PCR) amplicon sequencing of a portion of prophage regions, which were shown to be conserved among isolates from the same facility [7].

Whole-genome sequencing (WGS) has expanded the molecular surveillance from not only verifying the association between the patients and the food sources but also discovering evolution among isolates within the same or different clones. Further development and wide implementation of next-generation sequencing have greatly facilitated our understanding of genomic changes during evolution. Comparative genomics and evolutionary analysis on a small portion of prophage regions next to the phage insertion sites of the 1998 and 2002 *Lm* ECII outbreak isolates indicated that the prophage region might be conserved among isolates that were associated with the same outbreak, which represented a short-term evolution scenario [7,8]; these studies did not review the entire prophage regions. A similar phenomenon was observed in isolates from ECs associated with other listeriosis outbreaks that were linked to different types of foods when the entire prophage regions of these isolates were analyzed [9]. Thus, prophage regions might be important genetic markers to determine epidemiological relatedness and to identify resident strains. In addition to the prophage, a plasmid harbored by *Lm* ECII isolates associated with the 1998 listeriosis outbreak was characterized based on PCR amplicon and shotgun sequencing [10]. The PCR amplicon sequencing, targeting known resistance genes, such as *bcrABC* or *tmr,* conferring resistance to benzalkonium chloride (BC) and other disinfectants, was used to characterize the plasmid found in the 1998 U.S. outbreak isolates [11,12]. The absence of the complete prophage and plasmid sequences in these previous studies has prevented an evaluation of whether the MGEs also exhibit sufficient variations among strains to enable high resolution mapping of source populations.

Understanding the short-term evolution that occurred within the same clone may help shed light on the ability of *Lm* to persist despite environmental stresses. For example, *Lm* strains harboring *comK* prophages were shown to have greater persistence and rapid adaptation by biofilm formation on the food processing facilities, increasing the risk of repeated contamination [7]. Plasmids may similarly contain genes encoding important stress resistance features, such as resistance to BC and other disinfectants widely used in the food processing facilities [13]. The persistent strain(s) might have obtained resistance genes via horizontal genes transfer, which has been shown to occur through the transmission of mobile genetic elements (MGEs) [14]. Plasmid-borne resistance genes, such as *bcrABC* which confers resistance to BC, were previously reported on a putative transposon harbored by a plasmid of H7550, a 1998 listeriosis outbreak isolate, suggesting evidence of a role for MGEs contributing to adaptation and persistence of *Lm* strains in the meat or poultry processing facilities [12]. The gene cassette, *bcrABC*, was also observed in plasmids of multiple isolates from a 2017 outbreak associated with ice cream in Florida, but the cassette was not observed in plasmids of strains from other listeriosis outbreaks analyzed in that study [9]. Other plasmid-borne resistance genes, such as *qacA, qacC, emrE,* and *emrC,* were observed in plasmids of *Lm* strains isolated from the retail environment in the U.S. [15,16].

*Lm* prophages and plasmids are often larger than 30 Kb and include multiple repetitive regions [17,18]. As a result, shotgun sequencing and assembly based on short sequence reads (≤300 bp) cannot accurately distinguish these repeat sequences and collapse the entire MGEs [19]. The de novo assembly of shotgun sequencing then produces incomplete and fragmented genomes, thus, it may constrain us from capturing the entire MGEs sequences [19,20]. Recently developed long-read sequencing generating reads spanning the repetitive sequences enabled us to obtain a complete genome by closing gaps in fragmented assemblies [19,20,21]. However, compared with short-read sequencing, the assembled genomes obtained by long-read sequencing often showed a relatively high error rates, indicating assemblies based solely on Oxford Nanopore Technologies (ONT) may not accurately represent the true genome sequences [21]. To overcome the relative limitations of long-read and short-read sequencing technologies, both sequencing technologies can be combined, and a hybrid assembly pipeline can be used to generate complete genomes [22]. The objectives of the present study were to (i) employ long-read sequencing from ONT to close the genomes of *Lm* ECII isolates previously recovered up to 22 months apart from RTE meat or poultry processing environments, indicating possible persistence or repeated contamination of *Lm* [7], (ii) identify, characterize, and quantify variations among the complete MGEs based on WGS data of ECII isolates sequenced in this study and those previously sequenced isolates associated with listeriosis outbreaks that involved with RTE meat or poultry products, and (iii) assess the evolutionary rate of these ECII isolates for an improved interpretation of the population structure of persistent *Lm*.

## 2. Results

### 2.1. Complete Genomes Generated by ONT MinION and Illumina MiSeq Platforms

Seventeen isolates of *Lm* ECII recovered from seven RTE meat or poultry processing facilities (A, B, C, D, E, F, and G) in the U.S. between 2002 and 2009 (Table 1) were sequenced by Illumina MiSeq using the whole genome shotgun strategy; these isolates are not known to be associated with any outbreaks and thus were referred to as non-outbreak isolates hereinafter. Among them, five isolates, one each from five different facilities, were selected to be sequenced by ONT MinION. Upon successful sequencing of both Illumina and ONT, Unicycler used both in a hybrid assembly pipeline to generate a complete circular contig of the chromosome, with an additional circular contig if a plasmid was present (Table 2). Genome coverage of the complete chromosomes was 63.9× for OB020621, 18.5× for OB030029, 91.4× for OB040119, 86.7× for OB050226, and 38.4× for OB080183; the plasmid coverage was 54.3× for OB030029, 304.8× for OB040119, 166.8× for OB050226, and 60.0× for OB080183. After each genome was polished by its corresponding shotgun data, the total number of nucleotides changed was 35 for OB020621, 29 for OB030029, 25 for OB040119, 137 for OB050226, and 36 for OB080183.

### 2.2. Single Nucleotide Polymorphism (SNP) Analysis by Center for Food Safety and Applied Nutrition (CFSAN) SNP Pipeline

**Figure 1:**
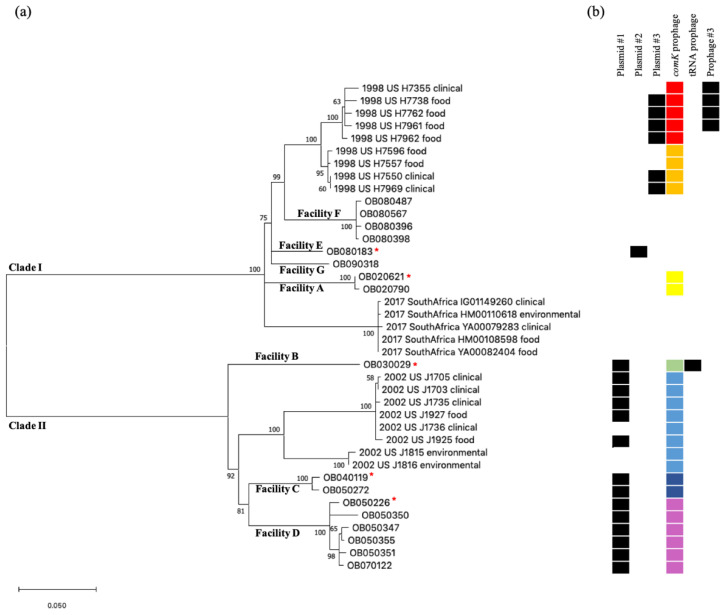
(**a**) Phylogenetic tree constructed by Center for Food Safety and Applied Nutrition (CFSAN) single nucleotide polymorphism (SNP) pipeline with bootstraps values (n = 1000) labeled on each branch. The isolates sequenced by Oxford Nanopore Technologies MinION are indicated (*). Isolates originated from the same facility or the same outbreak clustered together under one clade. (**b**) The presence or absence of each plasmid and prophage is indicated (presence: black/absence: blank). Identical *comK* prophages are indicated with the same color.

CFSAN SNP Pipeline was used to identify SNPs among the 17 non-outbreak isolates (Table 1) and 22 shotgun sequenced isolates from three different listeriosis outbreaks occurred in the U.S. and South Africa associated with RTE meat or poultry products (Table 3). The complete genome of OB020621 (Facility A) was chosen to be the reference for chromosomal sequences of these isolates. A phylogenetic tree was generated and isolates belonging to the same outbreak or the same processing facility formed monophyletic clades (Figure 1a).

The non-outbreak isolates from each cluster differed by less than 20 SNPs: the two isolates from Facility A differed by 2 SNPs; the two isolates from Facility C differed by 6 SNPs; the six isolates from Facility D showed difference by 4 to 20 SNPs (median, 10 SNPs); and the four isolates from Facility F differed by 1 to 4 SNPs. Therefore, isolates from each facility likely belonged to the same strain. The number of SNPs between clusters representing different facilities ranged from 45 to 247 SNPs.

Nine food and clinical isolates from the 1998 listeriosis outbreak were grouped into one clade containing two subclades: one subclade containing five isolates (H7355, H7738, H7762, H7961, and H7962) differing by ≤11 SNPs, and the other subclade containing four isolates (H7550, H7557, H7596, and H7969) differing by ≤3 SNPs. The isolates from the two subclades differed by 15 to 21 SNPs. Eight isolates from the 2002 listeriosis outbreak similarly formed a single clade containing two subclades, one subclade contained six food and clinical isolates that differed by ≤5 SNPs while the second subclade contained two environmental isolates (J1815 and J1816) that differed by 3 SNPs. The two subclades had 60 to 65 SNP differences. Lastly, the five isolates associated with the South African outbreak formed a single clade with ≤3 SNPs. Among these three outbreaks, the South African outbreak isolates were relatively close to the 1998 U.S. outbreak isolates, and they differed by 66 to 80 SNPs. In contrast, the 2002 U.S. outbreak isolates and the 1998 U.S. outbreak isolates differed by 227 to 249 SNPs.

Comparing the relationship among all ECII isolates analyzed in this study, the non-outbreak isolates from Facilities A, E, F, and G, the 1998 U.S. outbreak isolates, and the South African outbreak isolates formed Clade I (Figure 1a), and they differed by 43 to 83 SNPs; the isolates recovered from Facilities B, C, and D, and the 2002 U.S. outbreak isolates formed Clade II (Figure 1a), and they differed by 63 to 113 SNPs. These two clades differed by at 218 to 257 SNPs.

### 2.3. Identification and Characterization of Prophages from Shotgun and Complete Genomes

Among isolates from the seven facilities, isolates from Facilities A, B, C, and D had “intact” prophages on the shotgun genomes predicted by PHASTER (PHAge Search Tool–Enhanced Release). OB020621 and OB020790 (Facility A) each had one 41.2 Kb “intact” prophage; OB030029 (Facility B) had two “intact” prophages, 42.5 Kb and 43.1 Kb; OB040119 and OB050272 (Facility C) each had one “intact” prophage, 95.4 Kb and 80.9 Kb, respectively; OB050226 and OB050350 (Facility D) each had one 42.6 Kb “intact” prophage while OB050347, OB050351, OB050355, and OB070122 (Facility D) each had two “intact” prophages, 27.9 Kb and 31.1 Kb. Isolates from Facilities E, F, and G had no “intact” prophages predicted, and the predicted “incomplete” prophages were all in the middle of shotgun contigs, meaning these prophages were not a fraction of a prophage that was split into multiple incompletely assembled contigs. An intact, complete *comK* gene (609 bp) was present in the isolates from Facilities E, F, and G, confirming that no *comK* prophage was present in these isolates. Each one of the “intact” prophages of the isolates from Facilities A, B, C, and D was determined as a possible *comK* prophage if it contained at least one piece of the disrupted *comK* gene (423 bp or 189 bp). These results led us to subject one isolate from each of Facilities A, B, C, and D to long-read sequencing to obtain its complete genome and to identify the complete prophage region.

Among the five complete genomes of isolates from Facilities A, B, C, D, and E, OB020621 from Facility A had one “intact” prophage of 54.3 Kb; OB030029 from Facility B had two “intact” prophages of 43.1 Kb and 53.9 Kb; OB040119 from Facility C had one 45.8 Kb “intact” prophage; and OB050226 from Facility D had one “intact” prophage of 53.9 Kb. In contrast, OB080183 from Facility E did not have any “intact” prophage predicted. We then identified complete or disrupted *comK* genes in these genomes and found that except for one (43.1 Kb) of the two prophages in OB030029, all other “intact” prophages were *comK* prophages. The other one “intact” prophage (43.1 Kb) found on the OB030029 complete genome was the only non-*comK* prophage and we identified tRNA-Lys as the insertion site. There was no “incomplete” prophage predicted from any of the complete genomes.

We subsequently used the *comK* gene to modify the beginning and end positions of PHASTER-predicted prophage from complete genomes and the lengths were ~41K bp for all *comK* prophages (Table 1). The PHASTER-predicted prophage regions from complete genomes were always ~6 Kb to ~14 Kb longer than those prophage regions modified using the *comK* gene. PHASTER predicted two attachment sites (*attL* and *attR*) to determine the beginning and end position of a prophage, and the attachment sites were always slightly upstream or downstream of the disrupted *comK* gene. The NCBI annotation of these predicted regions showed the actual phage-like proteins were always between the disrupted *comK* gene locations.

For the four isolates from Facilities A, B, C, and D, the predicted “intact” prophage of a complete genome always corresponded to an “intact” prophage of the shotgun genome of the same isolate. However, the “intact” prophage predicted from the shotgun genome of OB040119 (Facility C, 95.4 Kb) was twice the size of its corresponding region predicted from the complete genome of OB040119 (45.8 kb), which was finally determined to be a 40.2 Kb *comK* prophage. Detailed sequence analysis revealed that the 95.4 Kb prophage from the OB040119 shotgun genome contained two identical copies of the 40.2 Kb *comK* prophage. We subsequently used SKESA to assemble these short reads and the same region contained only one copy of the 40.2 Kb *comK* prophage, confirming that SPAdes assembly had an error. When we used the disrupted *comK* gene to modify start and end positions of these “intact” prophages from SKESA-assembled shotgun genome, the final length was consistent with the *comK* prophage identified from its corresponding complete genome.

There were no “incomplete” prophages predicted from any of the five complete genomes. In contrast, a 22.8 Kb or 16.9 Kb “incomplete” prophage was predicted from each of the shotgun genomes of these five isolates and shared 100% sequence identity among them. This “incomplete” prophage was in the middle of a shotgun contig for all five isolates and the examination of protein annotations showed that there was no integrase and the total length of phage-like proteins was only 9.4 Kb. This 22.8 Kb or 16.9 Kb region was also present in the complete genomes, but it was not predicted as an “incomplete” or “intact” prophage when the complete genomes were analyzed by PHASTER. This was consistent with our approach of not considering “incomplete” prophage regions predicted in the middle of shotgun contigs.

We then compared the prophages predicted from isolates that were subjected to long-read sequencing with the isolates from the same facility that were only subjected to short-read sequencing and identified a few differences. Specifically, for the Facility D isolates, the 27.9 Kb “intact” prophage of four isolates (OB050347, OB050351, OB050355, and OB070122) corresponded to the ~41 Kb *comK* prophage identified in long-read and short-read sequenced OB050226. Detailed examination of sequences revealed that the 27.9 Kb “intact” prophage was at the end of a shotgun contig in all four isolates, indicating that the *comK* prophage might be split into multiple shotgun contigs in these four isolates. We subsequently identified another portion (~13 Kb) of this *comK* prophage in another contig of each isolate, and that region was predicted as an “incomplete” prophage. The other shotgun-sequenced isolate, OB050350, from Facility D had a 42.6 Kb “intact” prophage predicted on a single contig, which corresponded to the *comK* prophage of OB050226 and the actual length was also ~41Kb after we modified its start and end locations using the disrupted *comK* gene. Therefore, for Facility D, among the five isolates subjected only to shotgun-sequencing, the *comK* prophage was intact in one isolate and split into at least two contigs in the remaining four isolates. Thus, these results showed that the “intact” prophages predicted by PHASTER may not be the actual complete prophage when the predicted region was located at the end of a shotgun contig. Another difference was observed among isolates in Facility C. Similar to the shotgun genome of OB040119, the 80.9 Kb “intact” prophage in the shotgun genome of OB050272 contained two identical copies of ~41 Kb *comK* prophage identified from the complete genome of OB040119, and SKESA assembly from the short reads only showed one copy of the *comK* prophage, indicating that SPAdes shotgun genome assembly had an error.

**Figure 2:**
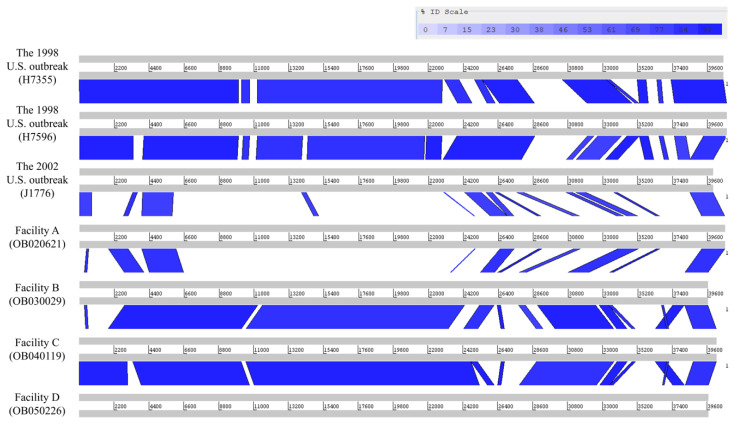
Artemis Comparison Tool comparison of seven complete *comK* prophages after modification using the disrupted *comK* gene. Each *comK* prophage contained ~65 genes in ~41 Kb regions. The two different *comK* prophages from the 1998 U.S. outbreak isolates shared 79% of genes. The *comK* prophage from the 2002 U.S. outbreak isolates shared higher percentage of genes with the H7355 *comK* prophage than H7596 *comK* prophage, 85% and 72% of genes, respectively. The *comK* prophages of Facilities A and B shared 22% of genes and the *comK* prophages of Facilities C and D shared 72% of the total number of genes.

In summary, the prophage prediction from the complete genome was slightly more accurate than those from the shotgun genomes because (i) shotgun genomes could have assembly errors caused by repeated regions in prophages, (ii) “incomplete” prophage predicted from shotgun genomes could be due to incompletely assembled fragments of an “intact” prophage and therefore inaccurate, and (iii) a prophage could be split into multiple shotgun contigs, resulting inaccurate predictions for both parts of the prophage. A total of four *comK* prophage regions were identified in the non-outbreak isolates from four facilitates (A, B, C, and D) (Table 1). Interestingly, isolates recovered from the same facility had a 100% identical (0 SNPs) *comK* prophage (Figure 1b). The four *comK* prophages from different facilities (A, B, C, and D) had significant sequence variations (Figure 2). The *comK* prophage of isolates in Facility A was most divergent from the other *comK* prophages of isolates from other facilities (i.e., 17% to 36% of BLAST alignment coverage (AC) with above 88% of sequence identity (SI)) (Figure 2). In contrast, the prophages from Facilities B, C, and D had a much higher similarity (i.e., 74% to 88% AC and 91% to 96% SI) (Figure 2). We also performed gene-by-gene BLAST comparisons; the four *comK* prophages contained 63 to 65 genes, the prophage from Facility A shared 14 to 30 genes with prophages from other facilities, and the prophages from Facilities B, C, and D shared 43 to 54 genes. These suggested that possible horizontal gene transfer or prophage replacement occurred during a short-term evolution.

### 2.4. Identification of Prophages Harbored in Lm Isolates from Three Outbreaks Associated with RTE Meat or Poultry Products

No complete genomes of the isolates from the 1998 U.S. outbreak were available in the NCBI database. PHASTER was used to predict prophages from genomes assembled with short-read shotgun sequencing data. The prophage profiles of the nine 1998 isolates corresponded to the two subclades of the phylogenetic tree generated by CFSAN SNP Pipeline (Figure 1). The first subclade contained five isolates, H7355, H7738, H7762, H7961, and H7962. PHASTER predicted a 43.5 Kb “intact” prophage and a 48.3 Kb “intact” prophage from both H7355 and H7738. The predicted prophages were all in the middle of relatively large contigs (i.e., 335 Kb and 620 Kb for H7355 and 195 Kb and 620 Kb for H7738, respectively), which increased our confidence in the prophage predictions from these shotgun genomes. The 43.5 Kb prophages from H7355 and H7738 were the same (100% AC and 100% SI) and harbored the disrupted *comK* gene near both ends. We subsequently modified it to be 40,606 bp using the disrupted *comK* gene. The 48.3 Kb prophages from H7355 and H7738 were also identical (100% AC and 100% SI), however, we could not determine the exact insertion site and referred to it as Prophage #3 hereinafter. PHASTER predicted a 41.1 Kb “intact” prophage at the end of a shotgun contig from each of H7762, H7961, and H7962. Only one part of the disrupted *comK* gene was found to flank this 41.1 Kb “intact” prophage region, and this 41.1 Kb “intact” prophage region was nearly identical to the 40,606 bp *comK* prophage (99% AC and 100% SI) from H7355 and H7388. Thus, we used the *comK* prophage from H7355 and H7388 as the reference and located the complete *comK* prophage from H7961, H7762, or H7962 that was split into two contigs. We subsequently determined that the *comK* prophages from all five isolates were identical (Figure 1b and Table 3). This is another example showing that the “intact” prophages predicted from shotgun genomes may not represent the complete prophage. Furthermore, PHASTER predicted prophage #3 in addition to the *comK* prophage from H7961 and H7762. H7962 did not have prophage #3, corresponding to the subclades of the phylogenetic tree (Figure 1).

The second subclade contained four isolates, H7596, H7550, H7557, and H7969. PHASTER predicted a 41.3 Kb “intact” prophage in the middle of a relatively long contig (i.e., 335 Kb) from H7596. We then identified the *comK* gene as the insertion site and used the disrupted *comK* gene to modify the prophage region to be 40,815 bp. PHASTER predicted a 29.7 Kb “intact” prophage at the end of a contig from H7550, H7557, and H7969 with identical sequences. This 29.7 Kb prophage was flanked by one part of the disrupted *comK* gene and partially aligned with the 40,815 bp *comK* prophage (72% AC and 100% SI). We then used the 40,815 bp *comK* prophage as the reference and identified the other part (~10 Kb) of this *comK* prophage from H7550, H7557, and H7969, which were predicted as “incomplete” prophages by PHASTER. The ~10 Kb was flanked by the second part of the disrupted *comK* gene and was always in a different contig from that containing the 29.7 Kb region. We subsequently determined that the *comK* prophages from each isolate within this subclade were identical (Figure 1b and Table 3).

The two *comK* prophages (40,606 bp and 40,815 bp) from the two subclades of the 1998 U.S. outbreak isolates had 81% AC to each other with 99% SI and they shared 51 genes out of the total of 65 genes (Figure 2), even though they were associated with the same outbreak. These suggested that possible horizontal gene transfer or prophage replacement occurred during a short-term evolution.

Four complete genomes from the 2002 U.S. outbreak isolates were available to increase the confidence of obtaining complete prophages with PHASTER analysis. All four complete genomes had one “intact” prophage predicted with the *comK* gene as the insertion site. We modified this prophage region to ~40 Kb using the disrupted *comK* gene. The eight shotgun genomes also had one “intact” prophage with the *comK* gene as the insertion site and there were no “incomplete” or “intact” prophages at the ends of a shotgun contig. The *comK* prophages predicted were identical (100% AC and 99% SI) in all complete and shotgun genomes (Figure 1b and Table 3).

PHASTER predicted one “intact” prophage of 62.2 Kb from the one complete genome (HM00113468) available for the South African outbreak. Two “intact” prophages of 62.2 Kb and 31 Kb were predicted in the middle of relatively large shotgun contigs (141 Kb and 148 Kb) from YA00079283 and we likely identified the complete region of each prophage. The 62.2 Kb “intact” prophages predicted from the complete genome and the shotgun genome corresponded to each other (98% AC and 97% SI) and this prophage was split onto multiple contigs in the other four shotgun genomes. We also identified a ~28.5 Kb “intact” prophage region and a ~18.8 Kb “incomplete” prophage regions that were at the end of shotgun contigs in all shotgun-sequenced isolates, and thus they could be only portions of prophage(s) and we could not identify the entire prophage(s). The 31 Kb, ~28.5 Kb, and ~18.8 Kb prophages were only predicted from shotgun genomes and they were not identified from the complete genome of HM00113468. Thus, gain or loss of prophages likely occurred in South African outbreak isolates. The ~18.8 Kb prophage region appeared to have tRNA-Lys as the insertion site, while the other three prophages (62.2 Kb, 31 Kb, and ~28.5 Kb) were not inserted into *comK* or near tRNA-Lys. All six South African isolates had an intact complete *comK* gene (609 bp) in their genomes, hence, there was no *comK* prophage integrated into the South African isolates.

### 2.5. Identification of Plasmids on Both Non-Outbreak and Outbreak Isolates

Out of the four plasmids identified by ONT MinION sequencing, the plasmids (hereinafter referred to as Plasmid #1) (~56 Kb) from OB030029, OB040119, and OB050226 (Facility B, C, and D, respectively) were 99% identical to each other by BLAST alignment. Comparison of Plasmid #1 with *Lm* plasmids in the NCBI database showed that it was nearly identical to the plasmid found in J1776 isolate of the 2002 U.S. outbreak (100% AC and 99% SI). The 90,421 bp plasmid (hereinafter referred to as Plasmid #2) found in the complete genome of OB080183 (i.e., the only isolate in Facility E) was different from Plasmid #1 (17% AC and 99% SI). Comparison with Plasmid #2 sequences deposited in NCBI showed that it had 98% AC and 99% SI to the plasmid of strain LM-F-75 (NCBI Accession number: KY613765, 91,243 bp).

BLAST of Plasmid #1 against 17 non-outbreak shotgun genomes from all facilities showed that this plasmid was present in the isolates belonging to Facilities B, C, and D (Table 1). This plasmid was split to 9 to 11 contigs in each shotgun genome. BLAST of Plasmid #2 against 17 shotgun genomes showed that it was only present in OB080183 (Table 1), split into five different contigs of its shotgun genome. Prediction by PlasmidFinder-2.0 and BLAST comparison against *Lm* plasmids deposited in the NCBI database did not identify additional plasmids in the 17 non-outbreak isolates.

**Figure 3:**
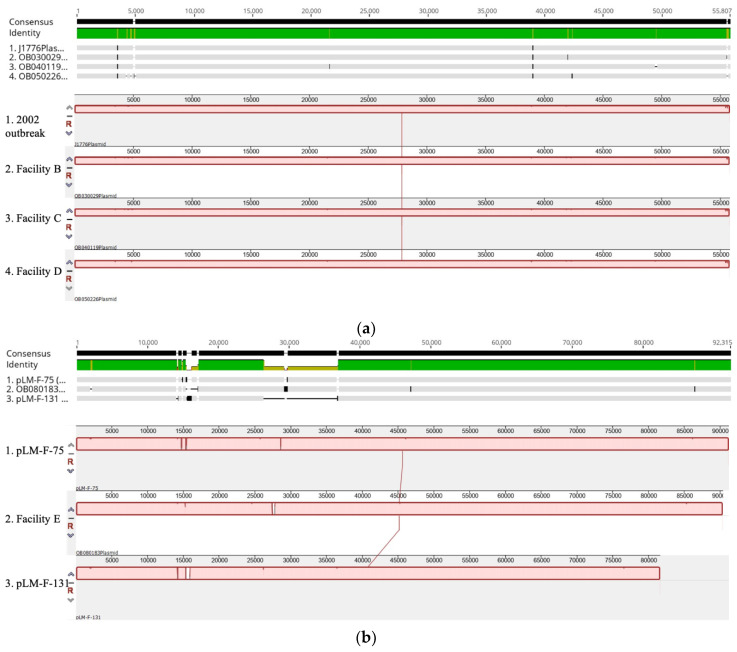
Mauve alignment of plasmids. (**a**) Plasmid #1: this plasmid (GenBank ID: CP006612.1) was found in J1776 of the 2002 U.S. outbreak isolates. The complete plasmid genome of Facilities B, C, and D isolates showed 100% alignment coverage (AC) and 99% sequence identity (SI) with pJ1776. (**b**) Plasmid #2 and #3: plasmid of LM-F-75 (GenBank ID: KY613765.1) from the NCBI database showed 98% AC and 99% SI to Plasmid #2 from OB080183 (Facility E). The strain LM-F-131 (GenBank ID: QADR01000015.1) has a plasmid with ~92% AC and 99% SI to Plasmid #3 found in the six shotgun genomes of the 1998 U.S. outbreak. The plasmid of LM-F-131 showed 100% of SI but 89% AC to Plasmid #2 (~90 Kb).

Among the four complete genomes of the 2002 U.S. outbreak (J1776, J1816, J1817, and J1926) deposited in NCBI, three except for J1816 had the same plasmid (~56 Kb, 100% AC and 99% SI among different isolates) which was nearly identical to Plasmid #1 (Figure 3a). Isolate J1816 did not have any plasmids (Table 3). Additionally, BLAST of Plasmid #1 against shotgun-sequenced isolates associated with this outbreak showed that five shotgun genomes (J1703, J1705, J1735, J1925, and J1927) harbored this plasmid (Table 3). The other two shotgun genomes (J1736 and J1815) did not have this plasmid. This was also consistent with PlasmidFinder-2.0 predictions and BLAST analysis compared with published *Lm* plasmids, which determined that J1736 and J1815 did not have any plasmids.

No complete genomes belonging to the 1998 U.S. listeriosis outbreak were deposited in the NCBI database. Among the nine shotgun genomes of isolates associated with the 1998 U.S. outbreak, six isolates were determined to harbor a plasmid by PlasmidFinder-2.0 and BLAST analysis. BLAST analysis of these shotgun genomes with Plasmid #1, Plasmid #2 and plasmids published in NCBI showed that all isolates harbored the same 82 Kb plasmid (hereinafter referred to as Plasmid #3), which aligned with the Plasmid #2 (~91 Kb) with 89% AC and 99% of SI (Figure 3b). Plasmid #3 also aligned with the plasmid of strain LM-F-131 (NCBI Accession number: NZ_CM009923.1, 81,666 bp) with ~92% AC and 99% SI. A benzalkonium chloride (BC) tolerance gene cassette, *bcrABC,* was found in both Plasmid #2 and #3.

There were no plasmids reported or found in the five shotgun genomes of the South African outbreak by PlasmidFinder-2.0. BLAST searches of the shotgun genomes against published *Lm* plasmids genomes in the NCBI database also could not find any plasmids within the genomes of South African outbreak isolates.

### 2.6. Tip-Dated Phylogenetic Analysis on Both Non-Outbreak and Outbreak Isolates Using Bayesian Evolutionary Analysis by Sampling Trees (BEAST)

**Figure 4:**
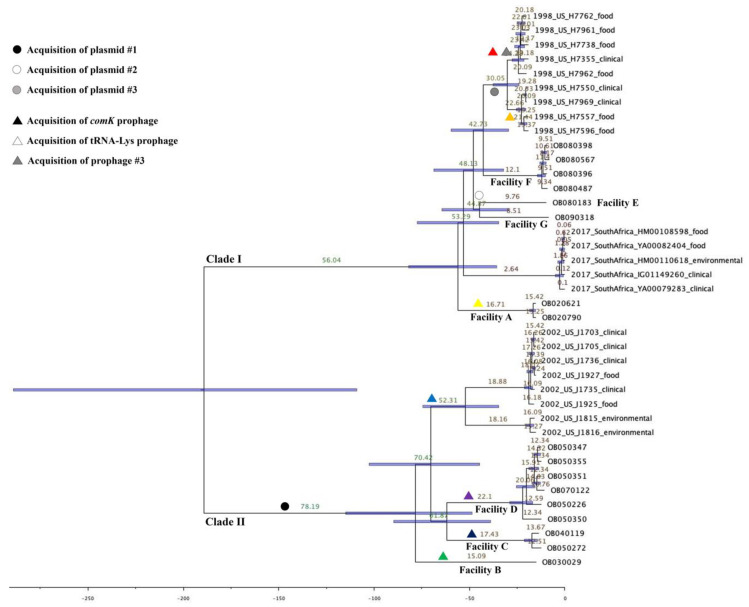
The maximum clade credibility (MCC) tree of 39 ECII isolates from Bayesian evolutionary analysis by sampling trees (BEAST) analysis. The mean heights were labeled on each branch and its 95% highest posterior density (HPD) was displayed in a horizontal bar. The possible acquisition events of plasmids and prophages are displayed; the color code of *comK* prophage matches Figure 1. The time of the most recent common ancestor (tMRCA) was estimated: (i) July 1968 (95% HPD, January 1961 to June 1974) for the 1998 U.S. outbreak isolates, (ii) February 1950 (95% HPD, January 1928 to November 1967) for the 2002 U.S. outbreak isolates, (iii) May 2015 (95% HPD, December 2012 to April 2017) for the South African outbreak isolates, and (iv) March 1816 (95% HPD, January 1716 to February 1896) for the entire 39 ECII isolates.

Based on marginal likelihood value and the effective sample size (ESS) values, the best fitting model for the 39 *Lm* ECII isolates was the strict clock model with coalescent constant population prior. The mean of substitution rate was 3.1 × 10^−7^ (95% highest posterior density, HPD, 1.6 × 10^−7^ to 4.6 × 10^−7^) nucleotide substitutions per site per year. The maximum clade credibility (MCC) tree (Figure 4) was generated from five independent runs and the time of most recent common ancestor (tMRCA) of various ECII isolates were estimated: (i) July 1968 (95% HPD, January 1961 to June 1974) for isolates from the 1998 U.S. outbreak, (ii) February 1950 (95% HPD, January 1928 to November 1967) for isolates from the 2002 listeriosis outbreak, (iii) May 2015 (95% HPD, December 2012 to April 2017) for five South African outbreak isolates, and (iv) March 1816 (95% HPD, January 1716 to February 1896) for the entire 39 *Lm* ECII isolates.

According to the MCC tree, the 39 isolates formed two major clades, Clade I and Clade II (Figure 4). The tMRCA for the Clade I containing the 1998 U.S. outbreak isolates, South African outbreak isolates, and eight non-outbreak isolates (Facilities A, E, F, and G) were estimated in March 1950 (95% HPD, March 1924 to May 1970). The other, Clade II was comprised of the 2002 U.S. outbreak isolates and nine non-outbreak isolates (Facilities B, C, and D) and the estimated tMRCA was in November 1925 (95% HPD, January 1889 to April 1955).

## 3. Discussion

Previous epidemiological studies have speculated that MGEs acquired by horizontal gene transfer may enhance resistance of *Lm* to disinfection and increase the risk of persistence or repeated contamination in food processing facilities [7,18,25,26]. These studies were based on shotgun and PCR amplicon sequencing data to determine specific genes or portions of MGEs because complete MGE sequences are difficult to identify by shotgun sequencing due to their large size and the presence of repetitive sequence elements. In recent years, long-read sequencing platforms have emerged and enabled us to obtain complete genomes [27]. Using a combination of both short-read and long-read sequencing platforms allowed us to generate accurate complete genomes with speed.

We employed ONT MinION and Illumina MiSeq sequencing platforms to analyze 17 *Lm* ECII isolates recovered from RTE meat or poultry processing facilities, not known to be associated with listeriosis outbreaks. All isolates were subjected to Illumina MiSeq sequencing and, based on MGEs analysis of shotgun data, five isolates were further subjected to ONT MinION sequencing. In addition, 22 shotgun sequencing data and five complete genomes of the isolates belonging to three ECII listeriosis outbreaks associated with RTE meat or poultry products were included to investigate the genetic relationship of *Lm* ECII strains that may have persisted in RTE meat or poultry processing facilities. The acquisition of MGEs via horizontal gene transfer could introduce highly variable sites with new genetic resistance features to *Lm* genomes during survival and growth in food matrix or on food processing environment [28]. Thus, MGE profiling could be relevant to differentiate closely related strains and also useful to trace the evolutionary relationship of *Lm* strains, especially within the same ECs, CCs, or strains that do not have many variations on their backbone genomes [28]. In our study, we focused on plasmids and chromosome-borne prophages to determine distinct genetic patterns of *Lm* ECII isolates associated with listeriosis outbreaks and isolates that might have been persistent or repeatedly contaminated RTE meat or poultry processing facilities in the U.S. We compared shotgun contigs to all plasmids published in the NCBI database and did not find any plasmids that might be integrated into chromosomes. The total length of plasmid contigs was the same as the length of the extrachromosomal plasmid identified by long-read sequencing. For the 1998 U.S. outbreak isolates, we do not have complete genomes, but the extrachromosomal plasmid of one of the outbreak isolates was isolated and characterized previously based on PCR amplicon sequencing and shotgun sequencing [12].

The complete sequences of *comK* prophage determined using the complete genomes could serve as references to help identify the presence or absence of each complete prophage region in shotgun genomes. Prophage predictions directly from shotgun genomes were mostly consistent with prophage predictions from complete genomes. Specifically, the *comK* prophages predicted from the shotgun genomes were mostly present in one contig, and corresponded to the disrupted *comK* gene that was in the same contig. We were able to modify the start and end positions of all PHASTER-predicted *comK* prophage using the the disrupted *comK* gene to obtain the exact prophage region encoding prophage-like proteins. However, in the case of the 1998 U.S. outbreak, no complete genomes were available, but in several isolates, the contigs containing the *comK* prophage were very long (e.g., 195 Kb, 335 Kb, or 620 Kb) which increased our confidence of correctly identifying the complete, intact *comK* prophage. Another inconsistent PHASTER prediction from the complete genomes and from the shotgun genomes was found on a few of Facility D isolates and the 1998 U.S. outbreak isolates. For four of the isolates associated with Facility D, the *comK* prophage was split into multiple contigs with two contigs containing 80% of the entire *comK* prophage and each contig containing one part of the disrupted *comK* gene (423 bp or 189 bp); one part of the *comK* prophage was predicted as “intact” and another part was predicted as “incomplete” by PHASTER. The remaining 20% of the *comK* prophage was split into at least three other contigs. For three of the isolates associated with the 1998 U.S. outbreak, the *comK* prophage was split into only two contigs with each contig containing one part of the disrupted *comK* gene; one part of the *comK* prophage was predicted as “intact” and the other part was predicted as “incomplete” by PHASTER. Lastly, the two non-outbreak isolates from Facility C showed inconsistent PHASTER prediction between shotgun and complete genomes due to the assembler error. Each shotgun genome of two non-outbreak isolates from Facility C assembled by SPAdes created two copies of the same prophage whereas SKESA allowed more accurate prophage predictions by PHASTER. This highlighted the value of using long-read sequencing since the completeness and an accuracy of the assembly had a remarkable effect on the correct identification of chromosome-borne prophages in bacterial genomes.

Among isolates that contained the prophages, prophage profiles and variations in prophages were consistent with the SNP-based phylogeny (Figure 1). Combining all non-outbreak isolates and outbreak isolates, the *comK* prophages among isolates from different outbreaks/facilities were significantly different (Figure 2), while they were generally conserved among isolates from the same outbreak/facility except for the isolates associated with the 1998 U.S. outbreak (Figure 1b) even though they were linked to a single food processing plant [29,30]. Our analysis identified two different *comK* prophages (81% AC and 99% SI) in the two subclades of isolates (15 to 21 SNPs) belonging to the 1998 U.S. outbreak with each subclade containing a mixture of food and clinical isolates. The two *comK* prophages shared 78% of genes (51 genes out of 65 genes). In addition, four out of five isolates in one subclade contained Prophage #3 of 48.3 Kb (Figure 1b). Interestingly, the four isolates containing Prophage #3 formed another subclade separated from the isolate (H7962) that did not contain Prophage #3 (Figure 1), showing consistency between the prophage variations/profiles and the phylogenetic clades of the 1998 U.S. outbreak isolates. Thus, we observed different *comK* prophage profiles among isolates associated with the same outbreak and linked to a single facility [30], and the difference was possibly caused by recombination, prophage gain, loss or replacement. This was not observed when small portions of the prophages from the same set of isolates were analyzed by PCR amplicon sequencing [7,8]. In contrast to the variations we observed among isolates associated with the 1998 U.S. outbreak, eight isolates associated with the 2002 U.S. outbreak separated into two subclades (60 to 63 SNPs) had the same *comK* prophage with no sequence variations. The two environmental isolates that formed a single clade only differed by 3 SNPs; they were recovered from two different food processing plants likely linked to the 2002 U.S. outbreak [5,31]. The 1998 and 2002 U.S. outbreak isolates were investigated using epidemiological evidence and pulsed-field gel electrophoresis (PFGE), and it is not surprising that isolates associated with each outbreak exhibited different prophage profiles or higher than average nucleotide diversity [3,23,32,33]. We also found that different isolates associated with the South African outbreak had different prophage profiles. The South African outbreak lasted for more than a year and analysis of additional isolates could shed more light on how prophages diversified and evolved during that time.

Prophages introduced a considerable amount of variation, possibly due to recombination and most of these variations, were removed before SNP-based phylogenetic analysis. Before filtering was applied in the CFSAN pipeline analysis of the 39 ECII isolates, there were 2266 variant sites (64.6% of the total number of variant sites) in the *comK* prophage region and 1240 variant sites (35.4%) in the backbone of the OB020621 chromosome. The CFSAN pipeline filtered high-density SNPs that might be introduced by recombination, and in this case, after filtering, 24 variant sites (3.5% of the total number of variant sites after filtering) were from the *comK* region and 662 variant sites (96.5%) were from the backbone of the chromosome. Thus, most of the variant sites filtered out were from prophage regions. Considering that, in general, prophages remained conserved among isolates associated from the same outbreak/facility and differed significantly among isolates from different outbreaks/facilities, including variations in prophages in the whole-genome comparison would likely result in consistent determination of phylogenetic relationship of *Lm* from these facilities. When we built a phylogenetic tree using the 3506 unfiltered variants sites (Supplementary Figure S1), isolates from each outbreak/facility were still clustered together. Nonetheless, since the variations from prophage regions could be caused by recombination, removing them from SNP-based analysis would avoid confusion, especially if the number of SNP differences will be used to help determine strain relatedness. In addition, the transmission of prophages is a horizontal genetic evolution, not following a pattern of vertical descent among shared ancestors; thus, although there is a close correspondence between MGEs and phylogeny, MGEs profiling should not be used directly for genetic clustering of isolates.

The isolates from three listeriosis outbreaks and 17 non-outbreak isolates were genetically dispersed throughout the representative *Lm* ECII strains submitted to the NCBI as of August 2019 (Supplementary Figure S2). The tMRCA for the 39 isolates was estimated in March 1816 (95% HPD, January 1716 to February 1896), 186 years before when we first recognized ECII strains in 2002 [4] with the average nucleotide substitution rate of 3.1 × 10^−7^ (95% HPD, 1.6 × 10^−7^ to 4.6 × 10^−7^) nucleotide substitutions/site/year. Previous studies have analyzed the average mutation rate on *Lm* strains using different strain collections. For example, the average substitution rate per year for 1696 *Lm* strains from both Lineages I and II is known to be about 2.6 × 10^−7^ (95% HPD, 1.9×10^–7^ to 3.4×10^–7^) substitutions/site/year [15], which was in the same range as our analysis. The mutation rate on 33 CC321 strains from lineage II was 1.15 × 10^−7^ (95% HPD, 0.79 × 10^−7^ to 1.52 × 10^−7^) substitutions/site/year [34]. In another study that calculated substitution rates among isolates associated with specific outbreaks, the average substitution rates were slightly higher, 5.5 × 10^−7^ to 5.8 × 10^−7^ substitutions/site/year for CC6 isolates associated with two different outbreaks linked to contaminated cheese products [9]. In our study, the time between the most recent common ancestor and the outbreak recognition was 30 and 52 years for the 1998 and 2002 U.S. outbreak, respectively. It was two years for the South African outbreak, and it was three and four years for the two previously analyzed cheese-associated outbreaks caused by ECII or CC6 isolates [9]. However, we have to interpret these results with caution because the selection of isolates would impact the estimate of the time of emergence. During the investigation of the 1998 and 2002 U.S. outbreaks, PFGE was used to determine strain relatedness, and thus strains with higher whole-genome diversity could have been chosen. We only obtained the genomes for a small portion of isolates associated with the South African outbreak and thus could underestimate the time of divergence of isolates associated with this outbreak.

The time of acquisition/loss of prophages and plasmids could be estimated with the evolutionary analysis using BEAST. In this study, we have identified seven different *comK* prophages; two from the 1998 U.S. outbreak isolates, one from the 2002 U.S. outbreak isolates and four from the isolates from Facilities A, B, C, and D. The *comK* prophage found in OB020621 and OB020790 of Facility A was likely to be acquired between March 1950 (95% HPD, March 1924 to May 1970), the estimated time of the divergence of Clade I, and January 1986 (95% HPD, March 1984 to May 1987), the estimated time of the divergence of OB020621 and OB020790 (Figure 4). The estimated tMRCA of the subclade of H7355, H7738, H7762, H7961, and H7962 was in April 1974 (95% HPD, October 1970 to January 1977) and that of the subclade of H7596, H7550, H7557, and H7969 was in December 1975 (95% HPD, February 1973 to February 1978). The estimated tMRCA of all nine isolates was in July 1968 (95% HPD, January 1961 to June 1974). Thus, the prophage unique to each subclade was possibly acquired between 1968 and 1974/1975. The *comK* prophage found in OB030029 (Facility B) could have been acquired after November 1925 (95% HPD, January 1889 and April 1955) when Clade II started to diverge (Figure 4). The isolates belonging to Facilities C and D had an estimated tMRCA in November 1943 (95% HPD, November 1915 to September 1966). The *comK* prophage found in Facility C isolates might have been acquired between November 1943 and July 1987 (95% HPD, September 1983 to August 1990) when the Facility C isolates started to diverge. The *comK* prophage in the six Facility D isolates might have been acquired between November 1943 and November 1983 (95% HPD, February 1977 to February 1989) when Facility D isolates started to diverge. Lastly, the 2002 U.S. outbreak isolates also harbored the identical *comK* prophage and it was likely to have been acquired between November 1925 and February 1950 (95% HPD, January 1928 to November 1967). The acquisition and loss events of the *comK* prophage could provide us an insight on the evolution of persistent *Lm* isolates in a food processing faciltiy.

Comparative analysis between BEAST analysis and plasmid profiling of 39 *Lm* isolates also enabled us to estimate the period of acquisition/loss of plasmids. Plasmid #1 was found in 14 isolates from Clade II (Figure 1). The plasmid was likely to be acquired before November 1925, when Clade II isolates started to diverge. Plasmid #2 was only found in OB080183 (Facility E), which might have been acquired in February 1964 (95% HPD, July 1944 and June 1979) when the isolates from Facilities E and G diverged. Then, this plasmid might have been lost in the isolates from Facilities G and F. In contrast, the 1998 U.S. outbreak isolates, which fell into the same clade as the isolates from Facilities E, F, and G, harbored Plasmid #3 which was genetically similar to Plasmid #2. Plasmid #3 shared a portion of Plasmid #2 found in OB080183 (89% of C, 100% SI), therefore, Plasmid #3 could have been evolved from Plasmid #2 after losing unnecessary genes before July 1968 (95% HPD, January 1961 to June 1974), the estimated tMRCA for the nine 1998 U.S. outbreak isolates. Plasmids #2 and #3 carried a BC tolerance gene cassette, *bcrABC*, which encodes proteins that help increase *Lm* tolerance against BC based disinfectants commonly used in the food processing facilities, and contributing *Lm* survival and persistence [11]. There was no plasmid among the South African outbreak isolates, but more than one prophage was conserved, which could be acquired before May 2015 (95% HPD, December 2012 to April 2017) when five isolates were likely to be diverged.

In summary, the complete genome offered by ONT sequencing enabled better identification of prophages and plasmids for subsequent sequence comparisons. Our analysis indicated that while shotgun genomes are still valuable for prophage prediction, there is still concern that shotgun genomes are not able to accurately resolve complete prophage regions. In addition, the entire plasmid would not be identified using only shotgun genomes assembled from short-read sequencing data. We showed that the MGE profiles and sequence similarities were concordant with the SNP-based phylogeny on *Lm* ECII isolates associated with three listeriosis outbreaks and isolates that might have persisted in meat or poultry processing plants. In addition, long-read sequencing technology allowed us to determine that in general prophage from the same facility or outbreak were conserved. This indicates that prophage sequences could serve as important genetic markers, allowing us to identify isolates of the same origin, especially during very recent short-term evolution. The identification and characterization of the MGEs based on a complete genome would provide accurate tracking information and effective molecular surveillance on the *Lm* strains that may be persistent in the food processing environments.

## 4. Materials and Methods

### 4.1. Isolates and Whole-Genome Sequencing

Seventeen *Lm* ECII isolates not associated with any listeriosis outbreaks were obtained from seven RTE meat or poultry processing facilities (A, B, C, D, E, F, and G) and sequenced by Illumina MiSeq (Table 1). Genomic DNA of each isolate was extracted from overnight cultures incubated at 37 °C in brain heart infusion broth (BHIB, Difco, Fisher Scientific, Canada) using QIAcube apparatus (QIAGEN Inc., Valencia, CA, USA) following the manufacturer’s manuals for Gram-positive bacteria. The library for Illumina short-read sequencing was prepared using a Nextera XT Library Preparation Kit (Illumina, Inc., San Diego, CA, USA). The sequencing was performed on an Illumina MiSeq platform with a 500-cycle Illumina MiSeq Reagent Kit v2 (Illumina, Inc., San Diego, CA, USA). Paired-end reads (2 × 250 bp) were trimmed using Trimmomatic v0.36.4 [35] with default parameters and de novo assembled using SPAdes v3.12.0 [36]. If our analysis on MGEs predictions indicated that SPAdes assemblies might contain errors, e.g., inconsistent predictions compared to the complete genome predictions, we used SKESA v0.24 [37] to assemble the raw reads again for analysis.

Among seven facilities, the shotgun genomes of isolates from Facilities F and G did not have MGEs predicted based on the analysis described in 4.3., while isolates from the other five facilities (A, B, C, D, and E) had MGEs predicted. Facilities C and E each had one isolate, which was subsequently subjected to long-read sequencing. One isolate of each of the Facilities A, B, and D was randomly chosen for long-read sequencing. A high-quality genomic DNA extraction was performed using Qiagen Genomic Tip 500/G columns (QIAGEN Inc., Valencia, CA, USA) per the manufacturer’s instructions. DNA library preparation for a long-read sequencing was followed using a 1D DNA ligation sequencing kit (SQK-LSK109) (Oxford Nanopore Technologies Inc., Oxford, UK) and DNA concentrations were measured using a Qubit dsDNA HS assay kit (Fisher Scientific Inc., Hampton, NH, USA). The prepared libraries were loaded into an ONT MinION flow cell (R9.4.1) and sequenced on the MinION device. The sequenced reads were base-called in real-time using MinKnow 3.4.8 integrated with Guppy 3.0.7. Long-read data were then assembled with corresponding shotgun data to obtain complete genomes [27] using a hybrid assembly pipeline in Unicycler v0.4.8 [38]. Short reads were first de novo assembled and long reads were used to close gaps and build bridges. Final complete sequences were polished multiple times using its short raw reads from Illumina MiSeq as a reference by Pilon v1.23 [39] included in Unicycler [38]. Genome coverage was calculated based on the total base pairs of initial raw reads divided by the final length of chromosome and plasmid.

Additionally, 26 publicly available *Lm* ECII isolates associated with three listeriosis outbreaks involving contaminated RTE meat or poultry products were selected for genomic comparisons. A total of 22 shotgun genomes and five complete genomes of the outbreak isolates were obtained from the NCBI database: nine isolates from the 1998 U.S. outbreak, 11 isolates from the 2002 U.S. outbreak and six isolates from the 2017–2018 South African outbreak (Table 3). The two environmental isolates associated with the 2002 U.S. outbreak, J1816 and J1815, were obtained from two different facilities. J1816 (Facility X) was likely linked to the outbreak and exhibited the outbreak PFGE pattern. J1815 did not have the same PFGE profile and it was isolated from a different facility (Facility Y) (Table 3) but it was considered to be associated with the 2002 U.S. listeriosis outbreak in the U.S. [5].

### 4.2. SNP-Based Phylogenetic Analysis

Whole-genome shotgun raw data of the 22 outbreak-associated isolates and 17 non-outbreak isolates were employed for phylogenetic analysis using the CFSAN SNP Pipeline v1.0.1 [40]. Both paired-end reads were aligned to the complete genome of OB020621 as a reference using Bowtie2 [41] and the variant sites were called using VarScan2 [42]. The reference genome OB020621 was chosen because it had no plasmids. The pipeline excludes high-density variant regions that contain at least three variant sites in any 1000 bp span because these variations might be caused by recombination events or an assembly error [43]. A SNP matrix was subsequently generated, which was used to construct a maximum likelihood tree with 1000 bootstraps values using MEGA X [44]. The SNP alignment of 39 isolates generated by CFSAN SNP Pipeline was further used for a tip-dated analysis described in 4.4.

### 4.3. Identification of MGEs: Plasmids and Chromosome-Borne Prophages

Based on previous studies suggesting that many of these ECII non-outbreak isolates contained *comK* prophages [7], we identified the *comK* gene (609 bp) from shotgun genomes to predict the possible presence of a *comK* prophage in a shotgun genome. The complete *comK* gene was identified in the one isolate from Facility E, all isolates from Facility F, and the one isolate from Facility G. Therefore, one isolate from four facilities (A, B, C, and D) was first chosen to be sequenced by ONT MinION for genome closure.

Putative prophages were predicted using PHASTER via a web portal [45] from all complete genomes and shotgun genomes. All “intact” prophages were subjected to further analysis to confirm the presence and completeness of prophage. “Incomplete” predictions found in shotgun genomes were considered only when the putative prophages were found at the end of a contig. A “questionable” prophage prediction of a 10.7 Kb length was observed in all isolates but not considered since it was previously described as monocin [10]. We then reviewed the genes in putative prophages, and also compared the locations of putative prophages and the *comK* gene. If a putative prophage was determined to be a *comK* prophage, we used the disrupted *comK* gene to modify its start and end locations. Then the complete sequences of identified *comK* prophages were compared using Artemis Comparison Tool v18.1.0 [46].

For the isolates of the 1998 U.S. outbreak from which no complete genomes were available, we performed PHASTER analysis on shotgun genomes and also aligned the *comK* gene to determine the possible presence of *comK* prophage. For the isolates of the 2002 U.S. outbreak and the 2017–2018 South African outbreak, we performed PHASTER analysis on complete genomes and shotgun genomes. If a putative *comK* prophage was identified, we modified its start and end locations using the disrupted *comK* gene.

After PHASTER analysis of individual genome, we then used BLAST: (i) to compare predicted prophages from different isolates, especially those predicted as “intact” prophages in the complete genomes and in the middle of shotgun contigs, (ii) to identify the prophage that was shared by all isolates or any prophage unique to specific isolate(s), and (iii) to establish if the prophages predicted are present in other complete or shotgun genomes.

We used PlasmidFinder-2.0 [47] for Gram-positive bacteria to predict possible plasmids, and also used BLAST (https://blast.ncbi.nlm.nih.gov) to compare shotgun genomes with all *Lm* plasmids published in the NCBI database to identify all plasmid contigs and to calculate the total length of each plasmid. We predicted fragments of plasmids from shotgun genomes in isolates from Facilities B, C, D, and E. There was no plasmid predicted from the isolates of Facilities A, F, and G. Thus, OB080183 from Facility E was additionally chosen to be sequenced by ONT MinION.

A discrete closed extrachromosomal sequence that was produced by the Unicycler hybrid assembler indicated the presence of a plasmid in that MinION-sequenced genome (Table 2). Subsequently, BLAST was used to search for those MinION-closed plasmids within the shotgun genomes that were assembled without nanopore sequencing data, with significant matches used to determine the presence/absence of these plasmids in shotgun genomes. The plasmids were then aligned with selected complete *Lm* plasmids published in the NCBI using Mauve alignment v1.1.1 [48] to determine if the plasmids were novel or previously reported from other *Lm* strains.

In the NCBI database, four out of eleven isolates associated with the 2002 U.S. outbreak isolates had complete genomes and of those, three contained plasmids. There were no plasmids reported for the 1998 U.S. outbreak isolates and the South African outbreak isolates, so we used BLAST analysis to compare their shotgun genomes against all *Lm* plasmids deposited in the NCBI database to identify possible plasmid contigs. We then calculated the total length of these plasmid contigs for identification.

### 4.4. Tip-Dated Phylogenetic Analysis Using BEAST

Among 39 genomes, the 686 SNPs were determined by the CFSAN SNP pipeline and the SNPs were used for a tip-dated phylogeny using BEAST v2.6.2 [49]. To find the best supportive model, three different clock models (strict clock, relaxed clock exponential, and relaxed clock log normal) were tested with three different priors: coalescent constant population, coalescent exponential population, and coalescent Bayesian skyline. For each run, the Hasegawa–Kishino–Yano (HKY) substitution model for nucleotide evolution was selected with four gamma categories and kappa parameter of 4.0. The Markov chain Monte Carlo (MCMC) was set to 100 million and the result (tracelog and treelog) was recorded every 2000 runs. Each combination was compared based on (i) the mean marginal likelihood values and (ii) the convergence with the effective sample size (ESS) value using Tracer v1.7.1 [50]. Then, five replicate runs of the best clock model and prior combination were performed, and the log and tree files were combined using LogCombiner v2.6.2 [49] with a 10% burn-in. TreeAnnotator v2.6.2 [49] was used to summarize the combined tree file as a maximum clade credibility (MCC) tree with common ancestor node heights and the tree was visualized using Figtree v1.4.4 (https://beast.community/figtree).

### 4.5. Accession Number(s)

Genomes of 17 non-outbreak *Lm* isolates sequenced in this study have been deposited in the NCBI/GenBank. Sequence read archive identifiers for draft genomes and accession numbers for five complete genomes were provided in Table 1.

## Figures and Tables

**Table 1:** Seventeen non-outbreak *Listeria monocytogenes* (*Lm*) isolates from ready-to-eat meat or poultry processing facilities in the U.S. including their sources, locations, dates of isolation, and the identified plasmids and the *comK* prophages with their lengths.

Isolate	Source	NCBI Accession Number/SRA	State	Facility	Date of Isolation	Plasmid (Kb)	*comK* Prophage (bp)
**Non-outbreak isolates** [7]							
OB020621 ^1^	Food	CP053478 /SRR12487739	NC	A	Sep-2002	NO ^2^	40,697
OB020790	Food	SRR12481282	NC	A	Nov-2002	NO	40,697
OB030029 ^1^	Food	CP053628-29 /SRR12487752	IN	B	Jan-2003	~56	39,610
OB040119 ^1^	Food	CP053630-31 /SRR12487754	PA	C	Jun-2004	~56	40,199
OB050272	Food	SRR12481281	PA	C	Aug-2005	~56	40,199
OB050226 ^1^	Food	CP053632-33 /SRR12487755	PA	D	Jul-2005	~56	39,677
OB050347	Environmental	SRR12481277	PA	D	Oct-2005	~56	39,677
OB050350	Environmental	SRR12481276	PA	D	Oct-2005	~56	39,677
OB050351	Environmental	SRR12481275	PA	D	Oct-2005	~56	39,677
OB050355	Environmental	SRR12481274	PA	D	Oct-2005	~56	39,677
OB070122	Environmental	SRR12481273	PA	D	May-2007	~56	39,677
OB080183 ^1^	Food	CP060526-27 /SRR12481272	NY	E	May-2008	~90	NO
OB080396	Environmental	SRR12481271	NJ	F	Aug-2008	NO	NO
OB080398	Environmental	SRR12481270	NJ	F	Aug-2008	NO	NO
OB080487	Environmental	SRR12481280	NJ	F	Oct-2008	NO	NO
OB080567	Environmental	SRR12481279	NJ	F	Dec-2009	NO	NO
OB090318	Environmental	SRR12481278	NY	G	Aug-2009	NO	NO

The National Center for Biotechnology Information (NCBI) accession number and/or sequence read archive (SRA) identifier for each isolate are included. ^1^ Complete genomes generated in this study. ^2^ NO, no plasmid or *comK* prophage was identified.

**Table 2:** The complete genomes of five non-outbreak isolates using both long-reads and short-reads generated by Unicycler assembler.

Isolate	Facility	Circular	Number of Contigs	Length of Chromosome (bp)	Length of Plasmid (bp)	Most Closely Related Plasmid in NCBI (99% Identity)	Position of the *comK* Prophage in the Chromosome
OB020621	A	Yes	1	2,949,231	NO ^1^	NO	2,365,689 to 2,406,385
OB030029	B	Yes	2	2,994,628	55,801	pJ1776	2,411,970 to 2,451,579
OB040119	C	Yes	2	2,953,956	55,803	pJ1776	2,370,708 to 2,410,906
OB050226	D	Yes	2	2,953,451	55,798	pJ1776	2,370,726 to 2,410,402
OB080183	E	Yes	2	2,908,728	90,421	pLM-F-131	NO

All five genomes were closed (circular) and OB020621 had one circular contig of chromosome, while each OB030029, OB040119, OB050226, and OB080183 had two circular contigs, a chromosome and a plasmid. For each plasmid that was identified from complete genomes, BLAST analysis was used to identify the most closely related *Lm* plasmid deposited in the NCBI database. The positions of each modified *comK* prophage on the complete genomes are included. ^1^ NO, no plasmid or *comK* prophage was identified.

**Table 3:** Twenty-six listeriosis outbreak-related isolates analyzed in this study.

Isolate	Source	NCBI Accession Number/SRA	State	Facility	Date of Isolation	Plasmid (Kb)	*comK* Prophage (bp)
**1998 U.S. isolates** [23]							
H7355	Clinical	SRR1814362	n/a ^1^	n/a	Nov-1998	NO ^2^	40,606
H7738	Food	SRR3707884	OH	n/a	Dec-1998	~82	40,606
H7762	Food	SRR3707885	MI	n/a	Nov-1998	~82	40,606
H7961	Food	SRR1814399	OH	n/a	Jan-1999	~82	40,606
H7962	Food	SRR1815438	OH	n/a	Jan-1998	~82	40,606
H7550	Clinical	SRR1815437	NY	n/a	Oct-1998	~82	40,815
H7557	Food	SRR3707886	NY	n/a	Nov-1998	NO	40,815
H7596	Food	SRR1815440	NY	n/a	Sep-1998	NO	40,815
H7969	Clinical	SRR3707879	OH	n/a	Jan-1998	~82	40,815
**2002 U.S. isolates** [23]							
J1776 ^3^	Clinical	NC_021839.1	NJ	n/a	Sep-2002	~56	39,947
J1816 ^3^	Environmental	NC_022047.1 /SRR2544677	PA	X	Oct-2002	NO	39,928
J1817 ^3^	Environmental	NC_021830.2	PA	X	Oct-2002	~56	39,932
J1926 ^3^	Food	NC_021840.1	PA	n/a	Nov-2002	~56	39,947
J1703	Clinical	SRR3707894	PA	n/a	Sep-2002	~56	39,947
J1705	Clinical	SRR3707893	PA	n/a	Sep-2002	~56	39,947
J1735	Clinical	SRR3707726	n/a	n/a	Jan-2002	~56	39,947
J1736	Clinical	SRR1815439	PA	n/a	Sep-2002	NO	39,947
J1815	Environmental	SRR3707728	MI	Y	Jan-2002	NO	39,932
J1925	Food	SRR1814333	n/a	n/a	Nov-2001	~56	39,947
J1927	Food	SRR3707892	PA	n/a	Nov-2002	~56	39,947
**2017–2018 South African isolates** [24]							
HM00113468 ^3^	Food	NZ_CP058256	n/a	n/a	Feb-2018	NO	NO
HM00108598	Food	SRR7056256	n/a	n/a	Jan-2018	NO	NO
HM00110618	Environmental	SRR7056247	n/a	n/a	Feb-2018	NO	NO
IG01149260	Clinical	SRR7056255	n/a	n/a	Dec-2017	NO	NO
YA00079283	Clinical	SRR7056251	n/a	n/a	Dec-2017	NO	NO
YA00082404	Food	SRR7056250	n/a	n/a	Jan-2018	NO	NO

The NCBI accession numbers for the complete genomes and SRA identifiers for the draft genomes are included. The plasmids and *comK* prophages determined in this study are shown with their lengths. ^1^ n/a, metadata not available. ^2^ NO, no plasmid or *comK* prophage was determined. ^3^ Complete genomes available in the NCBI database.

## Supplementary Materials

The following are available online at https://www.mdpi.com/2076-0817/9/10/822/s1. Figure S1: phylogenetic tree constructed by CFSAN SNP pipeline without filtration. The complete genome of OB020621 was used as a reference genome. The bootstraps values (n = 1000) were labeled on each branch. Figure S2: maximum likelihood tree of 17 non-outbreak isolates (in red) with 354 representative Listeria monocytogenes ECII strains from the NCBI database. Table S1: summary of PHASTER reports of the 39 shotgun genomes of Listeria monocytogenes ECII isolates included in this study.
