# Supplementary figures and images for: Characterization of Mobile Genetic Elements Using Long-Read Sequencing for Tracking Listeria monocytogenes from Food Processing Environments
Source: Pathogens. 2020 Oct 7;9(10):822. doi: 10.3390/pathogens9100822 (PMC7599586; doi:10.3390/pathogens9100822)

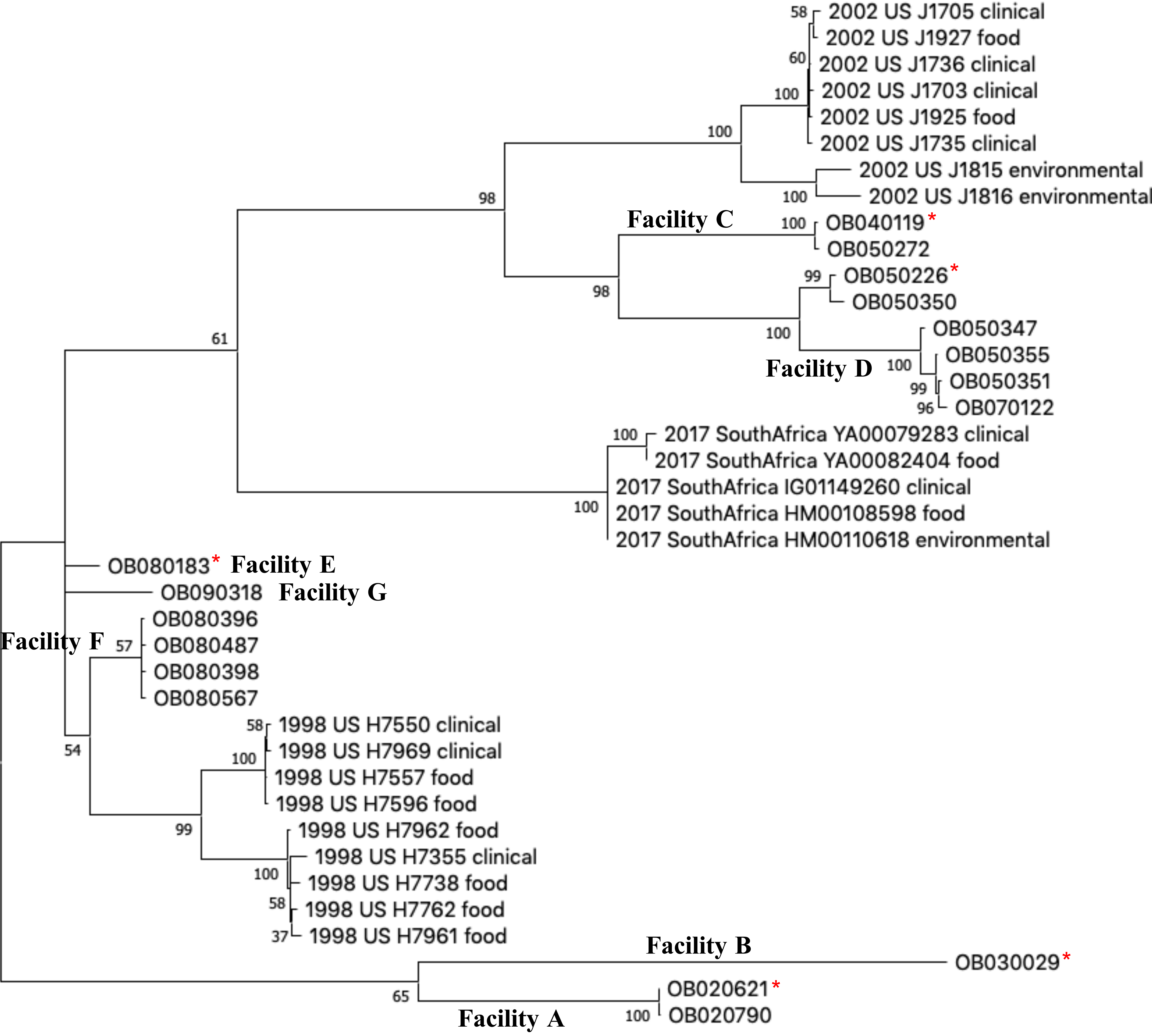

0.10

Supplement: Supplementary file 1 [file pathogens-09-00822-s001.zip › Supplementary materials/SuppFig1.pdf]

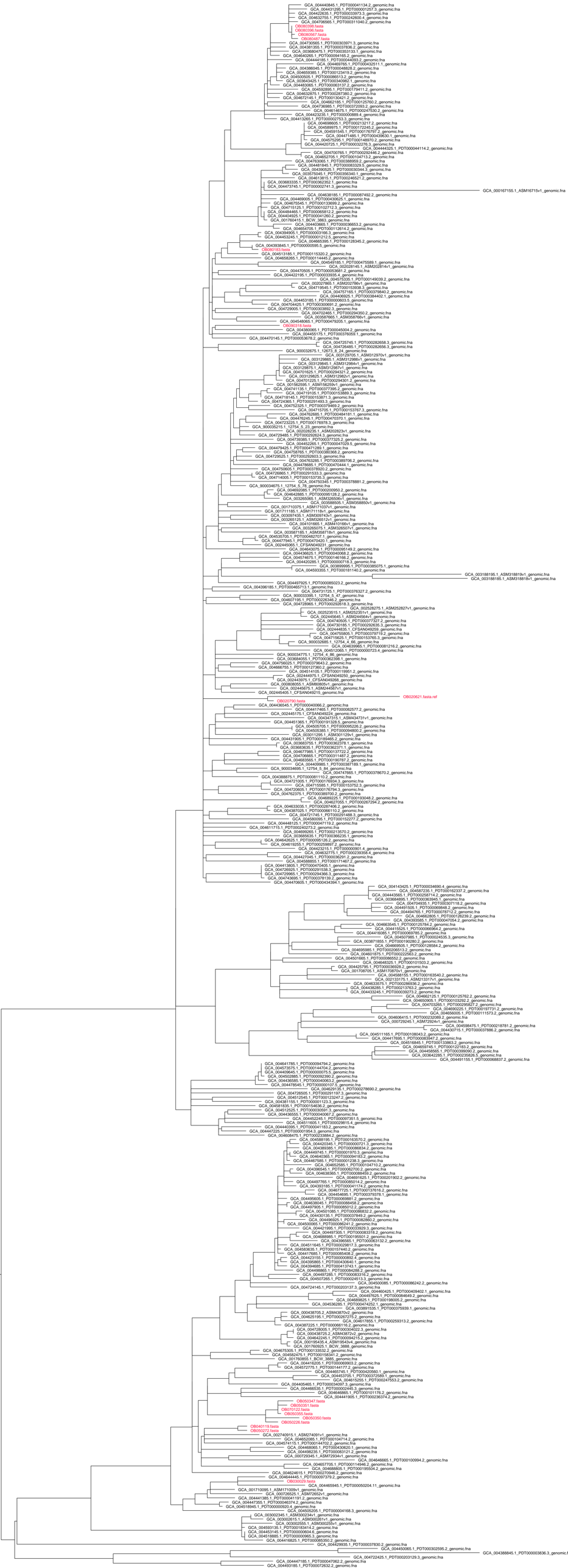

Supplement: Supplementary file 1 [file pathogens-09-00822-s001.zip › Supplementary materials/SuppleFig2.pdf]
